# Aphid polyphenisms: trans-generational developmental regulation through viviparity

**DOI:** 10.3389/fphys.2014.00001

**Published:** 2014-01-24

**Authors:** Kota Ogawa, Toru Miura

**Affiliations:** Laboratory of Ecological Genetics, Graduate School of Environmental Science, Hokkaido UniversitySapporo, Japan

**Keywords:** *Acyrthosiphon pisum*, aphid, hemimetabolous insect, hormonal regulation, maternal signal, reproductive polyphenism, viviparity, wing polyphenism

## Abstract

Polyphenism, in which multiple discrete phenotypes develop from a single genotype, is considered to have contributed to the evolutionary success of aphids. Of the various polyphenisms observed in the complex life cycle of aphids, the reproductive and wing polyphenisms seen in most aphid species are conspicuous. In reproductive polyphenism, the reproductive modes can change between viviparous parthenogenesis and sexual reproduction in response to the photoperiod. Under short-day conditions in autumn, sexual morphs (males and oviparous females) are produced parthenogenetically. Winged polyphenism is observed in viviparous generations during summer, when winged or wingless (flightless) aphids are produced depending on a variety of environmental conditions (e.g., density, predators). Here, we review the physiological mechanisms underlying reproductive and wing polyphenism in aphids. In reproductive polyphenism, morph determination (male, oviparous or viviparous female) within mother aphids is regulated by juvenile hormone (JH) titers in the mothers. In wing polyphenism, although JH is considered to play an important role in phenotype determination (winged or wingless), the role is still controversial. In both cases, the acquisition of viviparity in Aphididae is considered to be the basis for maternal regulation of these polyphenisms, and through which environmental cues can be transferred to developing embryos through the physiological state of the mother. Although the mechanisms by which mothers alter the developmental programs of their progeny have not yet been clarified, continued developments in molecular biology will likely unravel these questions.

## Introduction

In an attempt to understand the basis for the morphological diversity observed in organisms, studies focusing on developmental regulation and the evolution thereof have become a central theme in modern biology. In some organisms, the surrounding environment can switch the developmental fate as well as act on phenotypes as selective pressures. “Phenotypic plasticity” refers to the emergence of phenotypic variation in a developing organism in response to changes in environmental conditions (Hall, 1999; West-Eberhard, 2003). An extreme example of phenotypic plasticity is “polyphenism,” in which discrete alternative phenotypes are produced from the same genotype in response to extrinsic factors (Nijhout, 1999, 2003). How a single genotype can produce such markedly different phenotypes is an important question in developmental biology. In polyphenism, developmental trajectories for alternative phenotypes can be illustrated using forked pathways with a developmental switch leading to alternative phenotypes (Nijhout, 1999). In many cases, the developmental switches are regulated by physiological factors, like hormones, that mediate environmental (or genetic) factors and differential developmental mechanisms (Nijhout, 2003; West-Eberhard, 2003). In all known cases of polyphenisms in insects, the switching mechanisms of developmental pathways leading to alternative phenotypes are mediated by either the timing of hormone secretion, the timing of a hormone sensitive period, or the threshold of hormone sensitivity (Nijhout, 1999).

Aphids, which are small insects belonging to the family Aphididae in the order Hemiptera (sucking bugs), are major agricultural pests that damage plants through ingesting plant sap for nutrition, and by transmitting viral diseases in many crops (Dixon, 1998). Of the approximately 5000 species of aphids that have been described to date (Aphid Species File, Version 5.0/5.0, http://aphid.speciesfile.org/), all employ apomictic parthenogenesis (clonal or asexual reproduction) as their primary mode of reproduction (Simon et al., 2002).

In addition to wing (winged/macropterous, brachypterous or wingless) and reproductive (sexual reproduction or parthenogenesis) polymorphisms (Miyazaki, 1987; Dixon, 1998; Le Trionnaire et al., 2008; Brisson, 2010), aphids also exhibit body-color and caste polymorphisms (Aoki, 1977; Miyazaki, 1987; Fukatsu, 2010; Tsuchida et al., 2010), most of which are also recognized as being polyphenisms. Many aphid species show wing polyphenism in which winged morphs appear in response to changes in environmental factors in order to facilitate migration to new host plants or habitats (Dixon, 1998; Braendle et al., 2006; Brisson, 2010). Reproductive polyphenism, in which sexual reproduction and parthenogenesis are switched depending on seasonal conditions, is also exhibited by many aphid lineages (Le Trionnaire et al., 2008; Davis, 2012), and in social aphids, caste polyphenism results in the production of soldier aphids which appear to defend their gall (nest) [Aoki, 1977; Hattori et al., 2013; reviewed in Itô (1989); Stern and Foster (1996); Shibao et al. (2010)]. Since these flexible phenotypes of aphids should contribute to the remarkable adaptations seen in aphids (Dixon, 1998), the characteristics of these diverse and plastic phenotypes are important in the areas of ecology, evolutionary biology, and developmental biology. Here we review the biological basis underlying polyphenism in aphids, with a particular focus on reproductive and wing polyphenisms, and consider the various developmental mechanisms underlying phenotypic changes in response to different environmental conditions.

## Reproductive polyphenism in aphids

### Adaptive significance of the reproductive polyphenism

Traditionally referred to as the “two-fold cost of sex,” asexual reproduction is generally considered to be useful for increasing population size at twice the rate that is possible by sexual reproduction as no males are produced (Williams, 1975; Maynard Smith, 1978). In addition, sexual reproduction may incur other costs, such as finding mates, which are avoided in animals that reproduce asexually. It may therefore not be surprising that reproduction by parthenogenesis has been acquired independently, and often secondarily, in numerous organisms (Schön et al., 2009).

**Figure 1:**
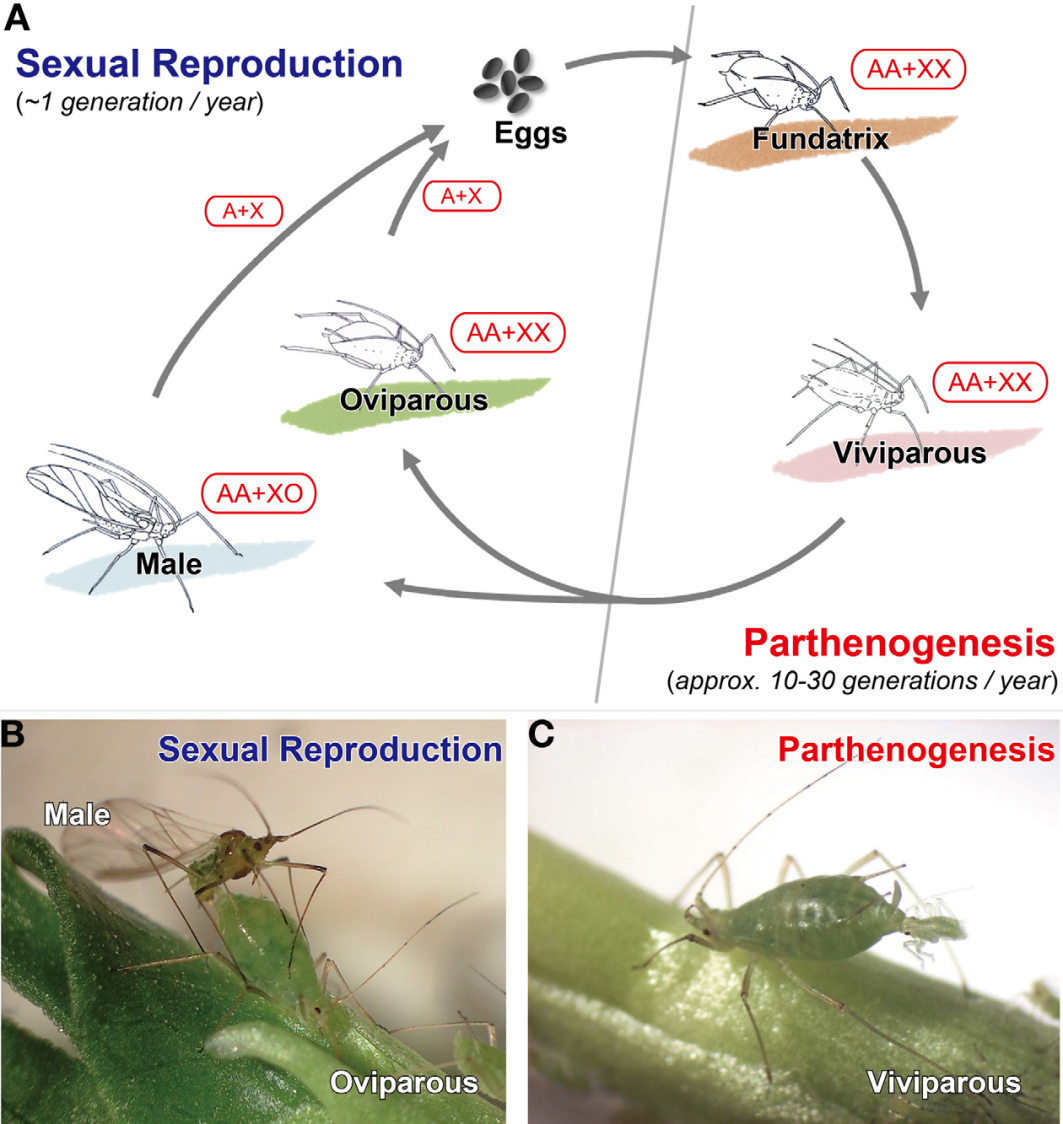
**Typical annual life-cycle of aphids**. **(A)** Schematic diagram of a typical holocyclic life cycle of aphids, **(B)** sexual individuals (male and oviparous female) of *Acyrthosiphon pisum*, **(C)** viviparous female of *A. pisum*. Aphids reproduce by thelytokous parthenogenesis in spring and summer under conditions of long day length and high temperatures. In aphids with a holocyclic life cycle, males and oviparous (sexual) females appear in late autumn and produce fertilized eggs for overwintering. Aphids employ the XO sex-determination system. Therefore, viviparous and oviparous females possess two X chromosomes, while males possess only one X chromosome. Males are produced parthenogenetically with the random loss of one X chromosome during the maturation division. Although oviparous females and males produce haploid oocytes and sperm, respectively, by reductive meiosis, only sperm possessing an X chromosome are viable (sperm lacking an X chromosome are degenerate). Therefore, the next generation, which will hatch as fundatrices in the spring, is entirely female (XX). Viviparous, viviparous parthenogenetic female; Oviparous, oviparous sexual female.

Aphids exhibit both sexual and asexual reproduction depending on the season. The typical annual life cycle of aphids consists of cyclical parthenogenesis which consists of a succession of parthenogenetic generations (approximately 10–30 generations in typical species) followed by a single sexual one (Moran, 1992; Simon et al., 2002; Figure 1). Many aphids with typical life cycles overwinter by employing frost-resistant, diapausing eggs, from which a female called a “fundatrix” or “stem mother” hatches in spring. These females are asexual and responsible for producing “viviparous females,” which also reproduce asexually until autumn. In late autumn, males and oviparous females, which are produced by parthenogenetic viviparous females, mate and lay overwintering eggs (Figure 1). Parthenogenesis in aphids is classified as apomixes, i.e., parthenogenesis in which the eggs do not undergo meiosis (Blackman, 1987). Consequently, except for spontaneous mutation and chromosome elimination in males (see *Chromosomal Sex Determination System in Reproductive Polyphenism* for a description of chromosome elimination in males), individuals within a single strain (lineage) are genetically identical (Blackman, 1987; Sloane et al., 2001; Davis, 2012). In the parthenogenetic oocytes of aphids, new centrosomes and microtubule-based asters, which are necessary for spindle formation in the first mitotic division, are organized spontaneously (Riparbelli et al., 2005); however, the centrosome is acquired from the male gamete during fertilization in sexually reproducing organisms (including sexual generation in aphids) (Riparbelli et al., 2005; Rodrigues-Martins et al., 2007).

**Figure 2:**
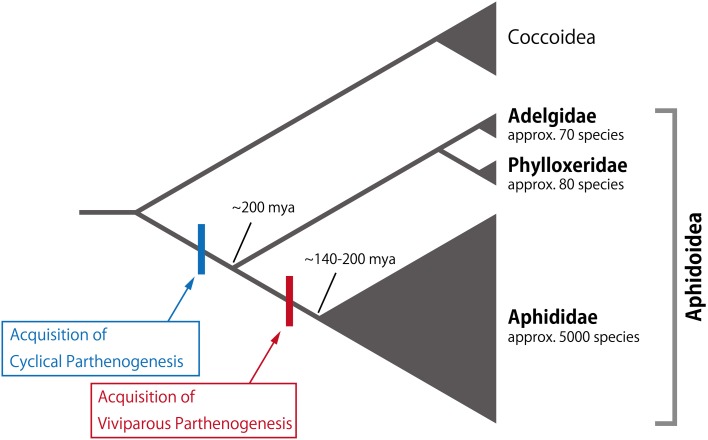
**Phylogeny of aphids and sister groups**. Cyclical parthenogenesis and viviparity are serially acquired in the Aphidoidea lineage. Extant species numbers in the families are indicated below each family name. Considerable diversification is seen in Aphididae, showing both of cyclical parthenogenesis and viviparity. The phylogeny and extant species numbers are based on Davis (2012) and Aphid Species File (Version 5.0/5.0, http://aphid.speciesfile.org/), respectively.

In the case of reproductive polyphenism, the induction of the sexual generation (males and oviparous females) and the subsequent production of eggs capable of surviving cold temperatures are a series of short-term adaptive responses to environmental cues indicating the onset of winter (Dixon, 1998; Simon et al., 2002, 2010). Although cyclical parthenogenesis is observed in all subfamilies in Aphididae (Simon et al., 2002; Figure 2), strictly asexual generations have only been described in approximately 3% of all aphid species (e.g., *Myzus ascalonicus* and *Toxoptera citricidus*) (Moran, 1992; Simon et al., 2002). In other words, the sexual generation may have been secondarily lost in these entirely asexual species. Moreover, approximately 30% of described aphid species include both cyclical-parthenogenetic and asexual clones (Moran, 1992; Dixon, 1998; Simon et al., 2002). Such strictly asexual species (or clones) tend to be distributed in low-latitude regions, suggesting that the sexual reproduction in aphids is an adaptation to severe winters, and that the parthenogenesis occurs in areas in which nymphal or adult aphids can overwinter (Simon et al., 1996, 2002, 2010; Rispe and Pierre, 1998; Rispe et al., 1998; Dixon, 1998). In other words, cyclical parthenogenesis in aphids has evolved in order to facilitate asexual viviparous reproduction as well as by overwintering by diapausing eggs (Simon et al., 1996, 2002, 2010; Rispe and Pierre, 1998; Rispe et al., 1998; Dixon, 1998). However, the above explanation cannot be applied to all species and groups. For example, in *Nipponaphis monzeni*, the sexual generation appears in early spring and laid fertilized eggs hatch in May of the same year (Kurosu and Aoki, 2009). Moreover, species belonging to Adelgidae and Phylloxeridae are capable of producing eggs through parthenogenesis, i.e., by oviparous parthenogenesis, indicating that sexual reproduction is not necessarily required for egg production (Granett et al., 2001; Havill and Foottit, 2007; Figure 2).

### Environmental cues responsible for switching reproductive modes

In many aphid species, short-day length and low temperature are major environmental cues for inducing the production of a sexual generation. The influence of photoperiod on the reproductive modes of aphids was first reported by Marcovitch (Marcovitch, 1923, 1924), who showed that sexual individuals of *Aphis forbesi* emerged in response to a short day length (Marcovitch, 1923); interestingly, that study was also the first report of photoperiodic induction in animals. In particular, in *Megoura viciae*, oviparous (sexual) females are produced when the day length is less than 14.5 h at 15°C; however, this response disappears above 23°C (Lees, 1959, 1963). Moreover, in *Acyrthosiphon pisum* which has a critical photoperiod of 13–14 h for the induction of sexual individuals (Lamb and Pointing, 1972), the critical photoperiod in the North American population increases by approximately 35 min for every 1°N latitude (Smith and MacKay, 1990). Aphids that produce sexual individuals perceive increases in night length (scotoperiod) from late summer to autumn (Hardie, 1990). In contrast, in some aphid species, sexual reproduction occurs in response to physiological changes of the host plants. For example, sexual morphs of *Aphis farinosa* and *Dysaphis devecta* are produced in response to cessation of host plant shoot growth (Forrest, 1970; Dixon, 1998). Furthermore, subterranean species living under constant darkness at relatively constant temperature also produce sexual morphs when the host plants become dormant (Dixon, 1998). However, it is not yet known which cues associated with the cessation of host-plant growth trigger these changes in aphids.

### Chromosomal sex determination system in reproductive polyphenism

Biologists have studied sex determination in aphids with complex life cycles for more than 100 years (e.g., Stevens, 1905, 1906, 1910; Morgan, 1909a,b). In addition to apomictic parthenogenesis, aphids employ the XO sex-determination system (Stevens, 1905, 1906; Wilson et al., 1997), which means that sexual females are genetically identical to their asexual mothers and, like their mothers, possess two X chromosomes. In contrast, males, which are hemizygous for the X chromosome (XO), are produced parthenogenetically with the random loss of one X chromosome during maturation division, which is modified meiosis without chromosomal reduction (Stevens, 1910; Morgan, 1915; Orlando, 1974, 1983; Blackman and Hales, 1986; Wilson et al., 1997; Figure 1). These sexual females and males then mate after producing haploid oocytes and sperm by the same reductive meiosis step employed by “normal” sexual organisms, but recombination then typically only occurs in the females (Blackman, 1976). In the males, only sperm possessing an X chromosome are viable, while sperm without X chromosome (O sperm) are degenerate (Stevens, 1905, 1906; Blackman, 1985). Consequently, the next generation, which hatches in spring, consists entirely of females (XX) (Figure 1). With the exception of several minor differences, this type of chromosomal manner is also found in adelgids and phylloxerans (Morgan, 1906, 1908, 1909a,b, 1912, 1915; Steffan, 1970; Havill and Foottit, 2007).

## Wing polyphenism in aphids

### Adaptive significance of wing polyphenism

The ability of insects to fly, which is considered to have been acquired only once in the class Insecta, has contributed enormously to their diversity and evolutionary success (Roff, 1990; Dudley, 2002). However, despite enabling insects to seek out new habitats, mates, and food resources, the capacity for flight is associated with considerable costs for insects (Harrison, 1980; Roff, 1990). Consequently, numerous insect species have secondarily lost the ability to fly in favor of allocating energy toward traits such as fecundity, longevity and weapons for intra- and interspecific competition (Harrison, 1980; Roff, 1990; Roff and Fairbairn, 1991). In other words, as a result of tradeoffs between flying ability and other traits, wing polymorphisms and/or flightless phenotypes (c.f. brachypterous or apterous/wingless) have evolved in numerous insect taxa (Wagner and Liebherr, 1992; Zera and Denno, 1997).

Wing polymorphism in aphids is associated with their complex life cycles (Brisson, 2010). In several aphid lineages (e.g., tribe Macrosiphini), wing polymorphisms have been attributed to both genetic and environmental factors, sometimes even within a single species (see *Wing Polyphenism and Genetic Wing Polymorphism* for details) (Smith and MacKay, 1989; Caillaud et al., 2002; Braendle et al., 2005a,b, 2006; Brisson, 2010). Viviparous females typically exhibit wing polyphenism and develop into winged or wingless morphs (Heie, 1987; Miyazaki, 1987; Dixon, 1998; Braendle et al., 2006; Brisson, 2010). In exceptional cases in the Drepanosiphinae and Phyllaphidinae, only oviparous females exhibit the wingless phenotype, while the other morphs are always winged (Heie, 1987; Dixon, 1998). In *Myzocallis kuricola*, though all viviparous females possess wings, dimorphism in the form of macropterous and brachypterous forms are known (Moritsu, 1983).

The winged and wingless phenotypes in aphids differ in a wide variety of morphological, physiological, life-history and behavioral characteristics. In addition to having wings and functional flight muscles, the fully winged morphs exhibit more extensive sclerotization of the head and thorax, more developed compound eyes, ocelli, longer antennae, more rhinaria, and occasionally larger siphunculi and cauda (Kalmus, 1945; Kring, 1977; Kawada, 1987; Miyazaki, 1987; Ishikawa and Miura, 2007; Ogawa et al., 2012). Most of these differences reflect the different lifestyles of the two morphs. For example, the winged morphs are equipped with an elaborate sensory system for flight and host plant location, and they are also more resistant to starvation (Tsuji and Kawada, 1987b; Hazell et al., 2005). In addition, the morphological differences between the winged and wingless phenotypes are usually correlated with differences in the ecological significance of each morph. Winged phenotypes typically have longer nymphal development periods, a longer pre-reproductive adult period, a longer reproductive period, lower fecundity, and prolonged longevity (Noda, 1960; MacKay and Wellington, 1975; Campbell and Mackauer, 1977; Tsuji and Kawada, 1987b; Tsumuki et al., 1990; Ogawa et al., 2012).

### Environmental cues responsible for switching between wing types

A number of environmental cues affecting the dispersal phenotypes (winged/macropterous or wingless/brachypterous) of aphids have been identified, especially in viviparous females (Hille Ris Lambers, 1966; Lees, 1966; Mittler and Sutherland, 1969; Müller et al., 2001). As in other insects, like locusts, density-dependent regulation of alternative dispersal phenotypes is widespread in aphids. Specifically, high-density triggers wing formation in many species (Johnson, 1965; Lees, 1967; Sutherland, 1969a; Shaw, 1970). Although the receptors of tactile stimuli have not yet been identified, the increase in tactile stimulation between individuals is considered to be the cue of high-density conditions (Johnson, 1965). In some species, antennae are considered to play an important role in the perception of tactile signals (Johnson, 1965; Lees, 1967; Sutherland, 1969a). The presence of natural enemies can also affect morph determination. In *Acyrthosiphon pisum*, winged-morph production is increased under conditions of high predation (Dixon and Agarwala, 1999; Weisser et al., 1999; Sloggett and Weisser, 2002; Kunert and Weisser, 2003). Two factors are considered to contribute to wing morph induction: the release of alarm pheromone, and the increase in tactile stimulation associated with avoidance behavior (Kunert et al., 2005, 2008). Conversely, the presence of ants that protect aphids from predators can inhibit both the induction of winged (dispersal) individuals and development of the flight apparatus (wings and flight muscles) (El-Ziady and Kennedy, 1956; Kleinjan and Mittler, 1975; Yao, 2012).

Traditionally, host plant quality (i.e., nutrition) was considered to be a key factor in morph determination (e.g., Sutherland, 1969b). However, Müller et al. (2001) showed that the findings of more than half of 38 studies on 12 different aphid species did not support the traditional hypothesis. In many of these earlier studies, morph determination appeared to depend on aphid density, although the density was considered to reflect the nutritive condition of the host plant (Müller et al., 2001). Nevertheless, in a few species, low nutrition alone can induce winged-morph production (Müller et al., 2001).

Furthermore, other factors, such as parasitoids, pathogens of aphids or plants, temperature, photoperiod, are also known to affect wing induction (White, 1946; Kenten, 1955; Johnson and Birks, 1960; Lees, 1966; Schaefers and Judge, 1971; Dixon, 1998; Müller et al., 2001; Leonardo and Mondor, 2006; Hatano et al., 2012). By responding to several of these stimuli rather than one, it is possible that aphids can track changes in environmental conditions more accurately. Indeed, it is likely that the multiple environmental stimuli that act on the central nervous system affect the physiology of the aphids, inducing wingless phenotypes possessing high fecundity under favorable conditions, and when conditions become unfavorable, allowing aphids to switch to the winged phenotypes to disperse to new habitats. In some aphids, such as *Megoura crassicauda* and *Acyrthosiphon pisum*, these environmental stimuli are processed by the mother aphid and the morphs of her resulting progeny are determined maternally or grandmaternally (Müller et al., 2001; Ishikawa and Miura, 2013). Thus, in these aphids, winged morphs are induced trans-generationally and if these aphid nymphs are crowded, the proportion of the winged adults in the same generation does not differ from other groups that have been reared under low-density conditions (Müller et al., 2001). However, in other aphid species, such as *Aphis craccivora* (Johnson, 1965), *Myzus persicae* (Sutherland and Mittler, 1971) and *Therioaphis maculata* (Toba et al., 1967), high density stimulus to younger nymphs can induce the winged morph in the same generation (Hille Ris Lambers, 1966; Lees, 1966; Müller et al., 2001). These findings suggest that the mechanisms underlying the developmental determination of wing polyphenism are specified (or optimized) in each aphid species or within groups.

### Wing polyphenism and genetic wing polymorphism

In the pea aphid, *Acyrthosiphon pisum*, several regulatory mechanisms are known to be involved in the wing polymorphisms/polyphenisms associated with the different reproductive modes observed in the annual life cycle of the aphid (Dixon, 1998; Braendle et al., 2006; Brisson, 2010). As in other aphid species, unfavorable environmental conditions can induce the expression of the winged phenotype in viviparous female generations (Lees, 1966; Sutherland, 1969a). On the other hand, wing polymorphism in males has a genetic basis and the *aphicarus* (*api*) locus on the X-chromosome is responsible for the determination of wing types (winged or wingless) (Smith and MacKay, 1989; Caillaud et al., 2002; Braendle et al., 2005a,b). However, all oviparous females and fundatrices are monomorphic wingless (Miyazaki, 1987; Brisson, 2010).

**Figure 3:**
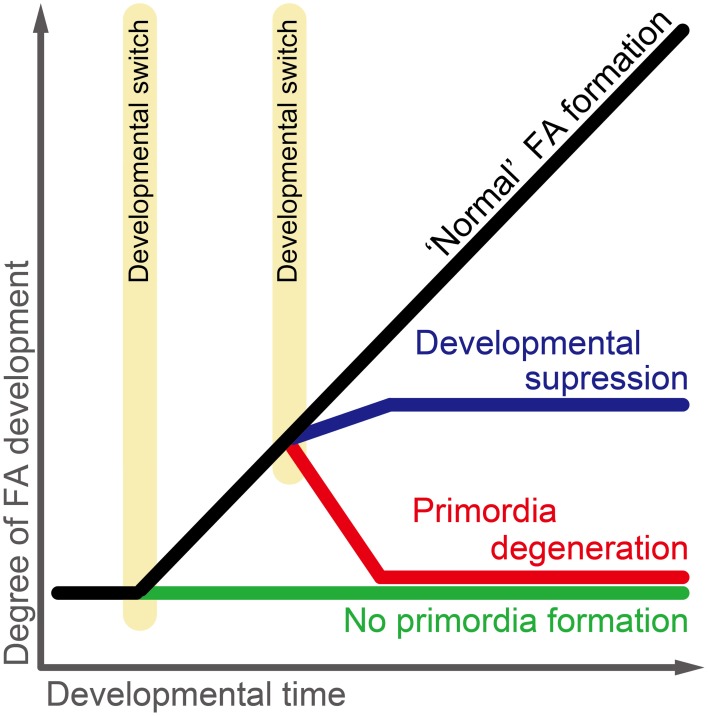
**Developmental trajectory of the flight apparatus (wings and flight muscle) in *Acyrthosiphon pisum***. Four developmental pathways and two developmental switches are hypothesized: the *normal flight apparatus formation pathway* is seen in wing and flight muscle primordia in winged males and viviparous females; the *developmental suppression pathway* is seen in flight muscle development of wingless males; the *primordia degeneration pathway* is seen in wing and flight muscle primordia development in wingless viviparous females and wing primordia development in wingless males; the *no primordia formation pathway* is seen in wing and flight muscle primordia development in fundatricies and oviparous females. Two developmental switches regulate primordia formation *per se* and the developmental fate of once-formed primordia, respectively. FA, flight apparatus.

Recent studies have shown that the development/degeneration processes of the flight apparatus (wings and flight muscle) differ among morphs of *Acyrthosiphon pisum* (Ogawa et al., 2012; Ogawa and Miura, 2013). Studies on female wing polyphenism showed that the first-instar nymphs of wingless viviparous females possess wing and flight-muscle primordia, which then degenerate during postembryonic development (Tsuji and Kawada, 1987a; Ishikawa et al., 2008). However, in the case of male wing polymorphism, the flight muscles of wingless morphs are developed and differentiated, even though they appear to be non-functional (Ogawa et al., 2012; Figure 3). Furthermore, the flight-apparatus primordia are not formed during embryogenesis or postembryonic development in oviparous females, or during postembryonic development in fundatrices (Ogawa and Miura, 2013; Figure 3). These findings suggest that although male and female winged forms both share similar developmental patterns, the regulation of flight apparatus development differs among wingless forms, which evolved secondarily from winged phenotypes. In other words, the diversity of derived developmental pathways in *Acyrthosiphon pisum* is considered to reflect the ecological traits of the respective morphs. For example, the pathway involved in primordia formation and context-dependent degeneration in viviparous females may facilitate a rapid response to environmental cues (Ogawa and Miura, 2013). Developmental regulation of the flight apparatus in *Acyrthosiphon pisum* is well suited for comparing and contrasting the developmental basis of genetically determined and environmentally induced phenotypes, as well as for considering how the evolutionary transition between genetic polymorphism, polyphenism, and monomorphism may have occurred.

## Physiological basis for aphid polyphenism

Endocrine factors, i.e., hormones have long been implicated in the control of polyphenism, either through spatial and temporal patterns of hormone levels or through the expression patterns of related factors in different tissues (Nijhout, 1999). For example, in the cricket genus *Gryllus*, a decrease in juvenile hormone (JH) esterase levels slows down JH degradation in the last nymphal instar. The relative increase in the JH titer compared to the ecdysteroid titer induces the development of short-winged morphs rather than of long-winged morphs (Zera et al., 1989; Zera, 2003). Similarly, in the honeybee *Apis mellifera*, workers feed those larvae designated to be queens relatively more royal jelly protein, which activates an endocrine response that elevates JH levels and eventually results in the production of queens rather than workers [reviewed in Hartfelder and Engels (1998)].

### Physiological basis for switching reproductive modes

Many aphids can accurately perceive scotoperiod length, and a specific number of constant dark- and light-cycles can induce sexual forms (Hardie, 1990). The perception of scotoperiod presumably employs an internal clock and a counting mechanism (Hardie and Nunes, 2001). The photoperiodic signal is detected directly by the brain through the cuticle or via the visual system (Lees, 1964; Le Trionnaire et al., 2009). It has been demonstrated experimentally that a group of neurosecretory cells (NSC) located in the dorsoanterior region of the protocerebrum (pars intercerebralis) was required for the perception of photoperiodic signals (Lees, 1964; Steel and Lees, 1977; Gao et al., 1999). Impairment of neurosecretory cells by cauterization inhibited the production of parthenogenetic morphs, even under short-night (long-day) conditions (Lees, 1964; Steel and Lees, 1977). Thus, it appears that sexual morphs are produced irrespective of photoperiod conditions when neurosecretory cells do not function, and physiological changes mediated by neurosecretory cells are required for reproduction of asexual morphs.

Although the capacity for parthenogenesis under short-night conditions would have been acquired by the ability of cells to secrete molecules that change the developmental fate of the oocytes, the transfer mechanism of the photoperiodic signal to the ovaries for orientation of the reproductive pathways (asexual or sexual) has not yet been resolved. However, it seems likely that such a transfer mechanism involves the endocrine system, e.g., melatonin or JH, at some stage (Corbitt and Hardie, 1985; Hardie et al., 1985; Gao and Hardie, 1997; Ishikawa et al., 2012). In particular, the JHIII titer in *Acyrthosiphon pisum* was shown to be lower in aphids producing sexual morphs under short-day conditions than in aphids producing parthenogenetic morphs under long-day conditions (Ishikawa et al., 2012). Furthermore, gene expression levels of JH esterase, which is responsible for JH degradation, were significantly higher in aphids reared under short-day conditions (Ishikawa et al., 2012). This suggests that up-regulation of the JH degradation pathway is responsible for the lower JHIII titer in aphids exposed to short days, leading to the production of sexual morphs. Global transcriptomic approaches have also detected changes in the expression of genes correlate with the response to photoperiodic cues (Le Trionnaire et al., 2009; Huybrechts et al., 2010). In particular, the expression of two genes in the insulin pathway is affected by decrease in day length: a transcript, probably coding for an insulin receptor, is down-regulated, and an enzyme transcript involved in insulin degradation is up-regulated (Le Trionnaire et al., 2009). This studies suggest that down-regulation of the insulin pathway occurred in the pea aphids reared under long-night (short-day) conditions (Le Trionnaire et al., 2009). In *Drosophila melanogaster*, the regulation of insulin pathway is correlated with the JH pathway (Tu et al., 2005). Furthermore, in *Culex pipiens*, the insulin pathway regulates diapause, which is also induced by decrease in day-length and changes in the relative concentration of JH (Sim and Denlinger, 2008). Similarly, the insulin pathway might contribute to the developmental regulation of reproductive polyphenism in aphids as an upstream regulator of the JH pathway (Huybrechts et al., 2010).

### Physiological basis for switching wing types

In the wing polyphenism of aphids, hormones are strong candidates for mediating the developmental responses to environmental cues. Although JH was previously considered to be a strong candidate for such a role, studies attempting to inhibit the production of winged progeny by manipulating JH titers have produced inconsistent results [reviewed in Braendle et al. (2006)]. In some aphid species, application of precocene II (PII), a plant-derived compound that is assumed to affect JH production, to mothers has been shown to induce the production of winged progeny [reviewed in Braendle et al. (2006)]. In other words, a decrease in the JH titer after PII application was considered to have induced winged progeny; however, subsequent studies have shown that this inducing effect was not mediated by JH. First, although PII has been shown to induce winged progeny in *Acyrthosiphon pisum*, PII was unable to induce precocious development, which is the classic benchmark for demonstrating the effect of decreased JH titers (Hardie et al., 1996). Secondly, co-application with JH fails to reverse the wing-inducing effects of PII (Gao and Hardie, 1996). Thus, the mode of PII action on wing induction is not mediated by JH and remains to be clarified.

Although JH has been shown to affect the development of the flight apparatus (Ishikawa et al., 2013), JH titers in parthenogenetic females subjected to high and low density conditions were similar (Schwartzberg et al., 2008), implying that JH is not responsible for fate determination leading to winged or wingless morphs. Alternatively, tissue-specific regulation downstream of the JH action may be important for fate determination, as well as the cases in other insects (Pursley et al., 2000; Parthasarathy et al., 2008). To clarify the relationship between JH and fate determination, comparative analyses across aphid species exhibiting differences in the induction mechanisms associated with wing development would be required, as the regulatory mechanisms may not be conserved completely among species.

### Trans-generational transfer of maternal signals through viviparity

Since the developmental fates of the various morphs are often determined prenatally, particularly insofar as reproductive and wing polyphenisms are concerned, the acquisition of viviparity by the Aphididae lineage likely constitutes the preadaptive basis for trans-generational regulation of these polyphenisms. The development of viviparity in aphids is considered to be an evolutionary innovation that may have contributed to the diversity and abundance of the Aphididae, which currently contains approximately 5000 species (thirty times more than the total number of species in Adelgidae and Phylloxeridae) (Davis, 2012; Figure 2). In viviparous parthenogenesis, the granddaughters of a female aphid (mother) are already developing within the daughter embryos of the mother. This nesting structure consisting of multiple generations has a high demographic potential, as considerable numbers of offspring can be produced asexually under favorable conditions. This type of viviparity in aphids is referred to as pseudoplacental viviparity.

**Figure 4:**
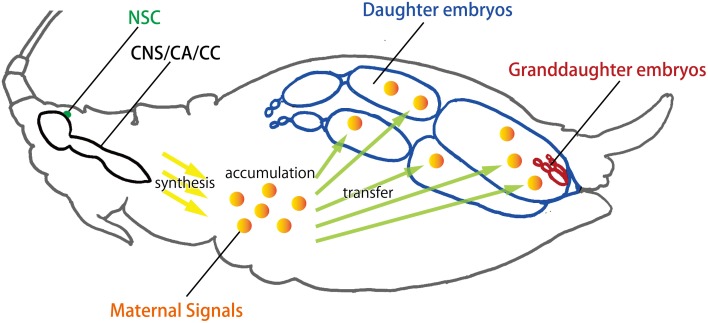
**Transgenerational transfer of maternal signals in viviparous aphids**. Viviparity in aphids is classified as pseudoplacental viviparity. Interestingly, the granddaughters of a female aphid (viviparous mother) are already developing within the daughters inside the mother. This telescoping association creates a unique opportunity for the mother to convey environmental information directly to her daughters (and granddaughters in some situations) via her hemolymph through the ovariole sheath. The multi-step events leading to the production of different aphid polyphenisms are hypothesized in this reproductive unit: (1) the mother perceives environmental information and integrates the information, probably via the NSC or CNS; (2) the mother converts the stimuli into a transmissible signal, probably in the CA or CC; (3) this signal is transmitted to the daughter embryos developing in the ovarioles of the mother; (4) the daughter embryos respond to the signal. CA, corpora allata; CC, corpora cardiaca; CNS, central nerve system; NSC, neuro secretory cells.

In pseudoplacental viviparity, nutritional input is provided by maternal hemolymph through the cells of the ovariole sheath, a unicellular epithelial layer that surrounds each ovariole [Couchman and King, 1980; reviewed in Bermingham and Wilkinson (2009)]. In addition to there being little or no yolk in the viviparous oocytes and embryos, there is also no chorion, probably because in addition to being dispensable, an eggshell could interfere with maternal provisioning of developing embryos (Blackman, 1987; Bermingham and Wilkinson, 2009). This close and continued association creates a unique opportunity for the mother to convey information about surrounding environments directly to her daughters (and granddaughters in some situations) before they are even born (Figure 4). In the case of oviparity, which is the method most widely found in insects, in order to convey environmental information transgenerationally, maternally-synthesized transmissible signals (e.g., mRNA) must be transferred to the eggs at the time of oviposition, which means that any such transfer of environmental information can only occur at the beginning of development. Furthermore, since developing embryos cannot move freely and do not have fully-differentiated and functional receptive organs, their perception of extrinsic factors might be limited. Taken together, the potential for environmental responsiveness associated with oviparity is likely to be lower than that associated with viviparity. Thus, the evolution of viviparity in aphids might provide the basis for rapid and flexible developmental switching.

Furthermore, viviparity might facilitate long-term environmental monitoring mediated by maternal or grand-maternal signals, as well as more reliable tracking (or prediction) of environmental conditions. For example, young clones founded by fundatrices produce no sexual individuals, even if they are reared under short-day low-temperature conditions (Lees, 1960; Dixon, 1972). This reproductive control is known as ‘interval timer’ and considered to be an adaptation to avoid the unexpected production of a sexual generation under short-day low-temperature condition in early spring (Lees, 1960; Dixon, 1972, 1998). Furthermore, winged adults and progeny descended from winged mothers rarely, if ever, produce winged offspring (Sutherland, 1970; Mackay and Wellington, 1977). However, wingless progeny derived from wingless mothers respond strongly to wing-inducing stimuli (Mackay and Wellington, 1977; MacKay and Lamb, 1979). These findings suggest that, in aphids, environmental information perceived by ancestors can be passed down through generations, and also that developmental fate must be determined following the integration of such successive information.

## Perspective: aphids as a model of polyphenism

As described above, the distinct polyphenisms observed in aphids could serve as model for the study of polyphenism in animals generally. Since the genome of *Acyrthosiphon pisum* has been sequenced is now open to public (International Aphid Genomics Consortium, 2010), *A. pisum* is the strongest candidate for such a model aphid species. Compared to other insect or arthropod genomes, the genome of *Acyrthosiphon pisum* is distinctive for several reasons (International Aphid Genomics Consortium, 2010). The genome is large (approximately 520 Mbp) and so is the number of predicted genes; more than 35,000 genes have been predicted based on sequence homology, EST, or RNA-seq data. The large number of genes is considered to be due to a large number of gene duplications (International Aphid Genomics Consortium, 2010). Specifically, 2459 gene families have undergone aphid lineage-specific duplications and more than 5 paralogs are contained in many families. Moreover, these paralogs account for almost half of the total number of aphid genes. The number of lineage-specific duplicated genes is greater than that of any other sequenced insect genome, although the genome of *Daphnia pulex*, which, like *A. pisum*, also exhibits extensive polyphenisms as well as has lineage-specific duplications (Colbourne et al., 2011). It is thus possible that multiple developmental pathways might be maintained in a single genome set as a result of these gene duplications. These aphid-specific gene duplications may also play a key role in polyphenic developmental regulation; for example, differential expression of diverged paralogs may occur in response to particular environmental conditions (Shigenobu et al., 2010). More precisely, environmental-dependent alternative expression of paralogous genes, which are newly acquired or which have lost a critical function, may determine the conclusive phenotype. This hypothesis is supported by numerous studies on differential paralogous gene expression between different morphs or between mothers that produce different morphs (e.g., Ramos et al., 2003; Cortés et al., 2008; Le Trionnaire et al., 2009; Brisson et al., 2010; Srinivasan et al., 2010; Lu et al., 2011; Price et al., 2011; Ishikawa et al., 2012; Duncan et al., 2013; Bickel et al., 2013).

Recently, epigenetic modifications to DNAs and to histones (e.g., methylation and acetylation) are considered to have contributed to the regulation of plastic developments in animals and plants (Bollati and Baccarelli, 2010; Blomen and Boonstra, 2011; Lyko and Maleszka, 2011; Feil and Fraga, 2012; Cortessis et al., 2012). In aphids, a functional DNA methylation system, functional small RNA system, and expanded sets of chromatin modifying genes were discovered as the factors related to the epigenetic developmental regulation [reviewed in Srinivasan and Brisson (2012)]. Interestingly, in *A. pisum*, a DNA region in a gene coding JH binding protein in winged viviparous females was highly methylated relative to wingless viviparous females (Walsh et al., 2010), suggesting the existence of regulatory cross-talk between physiological and epigenetic mechanisms. Therefore, future analyses of the coordinated regulation by epigenetic (e.g., methylation) and physiological (e.g., JH) systems will provide us new insight of the polyphenic development in aphids, in addition to the genomic regulation.

In addition to the availability of genome information, the ease with which the aphids can be reared and morphs can be induced by conditional manipulation is also an advantage of using *A. pisum* as a model organism. For example, induction systems for all *A. pisum* morphs have been established in our laboratory (Ishikawa et al., 2012; Ogawa et al., 2012; Ogawa and Miura, 2013). Furthermore, since all female phenotypes within the same strain are genetically identical, the influence of genetic variation can be ignored (Ogawa et al., 2012; Ogawa and Miura, 2013). To maximize the potential benefits associated with these characteristics, well-designed experiments that consider the unique developmental characteristics of aphid are required. For example, because aphids can respond to multiple environmental stimuli and transfer these environmental signals trans-generationally, pre-induction conditions in which aphids are reared under stable conditions through multiple generations before the onset of experiments is important. In our experience, such pre-induction has a marked improvement on experimental repeatability (Ishikawa et al., 2012). Moreover, strain selection is also important as induction rates have been observed to differ among strains, in both reproductive (Smith and MacKay, 1990; Hazell et al., 2005; Ogawa et al., 2012) and wing polyphenism (Lamb and MacKay, 1979).

On the other hand, *A. pisum* is an agricultural pest and techniques for gene function analysis in this species are not advanced. Sharing the same strains among members of the aphid research community would be straightforward due to their clonal reproduction. However, the importation of aphid strains is difficult and cumbersome procedures are required due to restrictions based on measures such as the International Plant Protection Convention (IPPC). From a molecular biological perspective, experimental procedures for techniques such as gene knockout/knockdown analysis in aphids have not yet been widely established; however, some studies on gene silencing by RNA interference (RNAi) have been published (Mutti et al., 2006; Jaubert-Possamai et al., 2007; Pitino et al., 2011; Mao and Zeng, 2012). Recently, genome-editing methods using zinc-finger nucleases (ZFNs) or transcription activator-like effector nucleases (TALEN) have been used extensively for genetic knockout in non-model insects (e.g., Watanabe et al., 2012). These techniques should also be applied to investigations on aphid polyphenism, providing new insights into the mechanisms and evolutionary processes associated with this phenomenon.

### Conflict of interest statement

The authors declare that the research was conducted in the absence of any commercial or financial relationships that could be construed as a potential conflict of interest.
